# Metabarcoding of *Chloraea* orchid roots reveals diverse fungal communities and a negative association between fungal diversity and geographic range

**DOI:** 10.3389/ffunb.2026.1870189

**Published:** 2026-07-10

**Authors:** Orlando Jeldes-Cajas, Janice Zúñiga, Alfredo Ceballos, Isadora Chávez, Guillermo Pereira, Marco A. Molina-Montenegro, Cristian Atala

**Affiliations:** 1 Laboratorio de Anatomía y Ecología Funcional de Plantas (AEF), Instituto de Biología, Pontificia Universidad Católica de Valparaíso, Valparaíso, Chile; 2 Centro de Investigación en Recursos Naturales y Sustentabilidad (CIRENYS), Facultad de Ciencias Médicas, Universidad Bernardo O’Higgins, Santiago de Chile, Chile; 3 Laboratorio Biotecnología de Hongos, Departamento de Ciencias y Tecnología Vegetal, Universidad de Concepción, Los Ángeles, Chile; 4 Centro de Ecología Integrativa, Instituto de Ciencias Biológicas, Universidad de Talca, Talca, Chile; 5 Centro de Investigación en Estudios Avanzados del Maule (CIEAM), Universidad Católica del Maule, Talca, Chile

**Keywords:** fungal community, metabarcoding, mycorrhizal specificity, orchid mycorrhizal fungi, terrestrial orchids

## Abstract

**Introduction:**

Orchid mycorrhizal fungi are essential partners for orchid seed germination and survival, yet the full diversity of fungi associated with orchid roots, including nonmycorrhizaltaxa, remains poorly characterized, particularly in the Southern Hemisphere. This could impact orchid conservation and *in situ* restoration programs. Here we present the first untargeted metabarcoding study of root-associated fungal communities in Chilean terrestrial orchids.

**Methods:**

We used ITS regsion for fungal metabarcoding, sampling eleven species of the genus *Chloraea* (Orchidaceae) across the central Chilean coast.

**Results:**

Root-associated communities were dominated by Ascomycota and were highly variable in composition among individuals and species, with orchid mycorrhizal fungi representing only a minor fraction of total diversity and the family Tulasnellaceae absent from all samples. Community composition differed significantly among *Chloraea* species, and contrary to expectations, species with broader geographic ranges tended to associate with less diverse root-associated fungal communities – a pattern that was consistent in direction across multiple diversity indices but sensitive to sampling effort. A substantial proportion of sequences could not be assigned taxonomically, reflecting the underrepresentation of Chilean fungal diversity in global reference databases.

**Discussion:**

These findings demonstrate the value of untargeted sequencing for revealing the breadth of orchid–fungi associations, and highlight that mycorrhizal specificity could vary markedly across *Chloraea* species, with direct implications for the conservation of threatened Chilean terrestrial orchids.

## Introduction

Orchidaceae is one of the most diverse families of flowering plants worldwide (Heywood et al., 2007; Chase et al., 2015; Christenhusz and Byng, 2016). Its diversity is concentrated mainly in tropical regions, although the family exhibits an almost cosmopolitan distribution (Givnish et al., 2015; Novoa et al., 2015). Orchids are highly relevant for biodiversity and conservation and they also hold economic and cultural value due to their use as ornamental plants and in the production of medicinal and food-related compounds (Hinsley et al., 2018). Despite their importance, orchids are among the most threatened plant groups as a result of land-use change, climate change effects, and unsustainable harvesting (Seaton et al., 2010; Atala et al., 2017).

Orchids are a highly specialized group characterized by complex biological interactions (Rasmussen, 2002), which represents an additional challenge for their conservation. Terrestrial orchids depend on orchid mycorrhizal fungi (OMF) to enable natural seed germination as well as the growth and survival of adult plants (Smith and Read, 2008; Pereira et al., 2021). This dependence arises because orchid seeds are extremely small and lack the reserves required for germination (Leake, 2004; Pereira et al., 2005; Dearnaley et al., 2016). At later stages, once plants reach maturity, the fungi receive carbon compounds produced by the orchid (Cameron et al., 2006; Smith and Read, 2008). This interaction is particularly relevant in partially or fully mycoheterotrophic species, whose survival strongly depends on mycorrhizal associations (Leake, 1994; Smith and Read, 2008).

The study of OMF has been conducted for more than a century (Rasmussen, 2002) and the nature of orchid–fungus associations varies widely among species and genera. Most identified taxa correspond to Basidiomycete fungi of the *Rhizoctonia* complex; specifically, Ceratobasidiaceae, Serendipitaceae, Sebacinaceae and Tulasnellaceae (Dearnaley et al., 2012). These associations range from highly specific to broadly generalist interactions involving multiple fungal symbionts (Otero and Bayman, 2009; Jacquemyn et al., 2011; Pereira et al., 2018; Li et al., 2021a; Mennicken et al., 2024). Generalist orchids may exhibit greater adaptive capacity under changing environmental conditions due to their interactions with a wider range of fungal partners (Swarts and Dixon, 2009). In contrast, specialist taxa may increase their biological fitness in specific habitats through associations with ecologically well-adapted fungi (Bonnardeaux et al., 2007). However, high mycorrhizal specificity can restrict ecological niches and geographic distribution when the availability or distribution of associated fungi is limited (Leake and Cameron, 2012). In this way, OMF influences local orchid abundance and population dynamics by facilitating adaptation to varying physiological demands and environmental conditions (McCormick and Jacquemyn, 2014; McCormick et al., 2018).

Reintroduction strategies involving ex situ–propagated orchids are more successful when suitable OMF are present at establishment sites (McCormick et al., 2018) and adult plants have a better overall performance when associated with OMF (Pereira et al., 2021). Although orchids may simultaneously associate with multiple fungi, in many cases the specificity of these interactions is highly restrictive, such that the absence of compatible fungal symbionts limits natural population regeneration (Rasmussen et al., 2015). Consequently, the identification of specific OMF and other types of mutualistic fungi such as endophytes (Herrera et al., 2019) represents a key tool in orchid conservation and restoration efforts (Fay, 2018; McCormick et al., 2018). Traditionally, studies have relied on culture-dependent methods and the identification of *Rhizoctonia*-associated mycorrhizae, which may underestimate the true diversity of fungi involved in these symbioses (Herrera et al., 2019; Mujica et al., 2020). In contrast, the application of molecular ecology tools, such as soil DNA metabarcoding and high-throughput sequencing technologies, has expanded our understanding of the diversity and structure of fungal communities associated with orchids, providing key information to strengthen conservation efforts (Martos et al., 2012; Jacquemyn et al., 2016; Nilsson et al., 2019).

The application of molecular tools has revealed that orchid roots harbor a highly diverse fungal community that extends well beyond canonical OMF. These non-mycorrhizal fungi, broadly termed orchid non-mycorrhizal fungi (ONF), are now recognized as common and consistent associates of orchid roots across tropical and temperate species, yet their functional roles remain largely unexplored (Ma et al., 2015; Li et al., 2021b; Watkinson and Winkel, 2024), opening up a new field of research related to orchid-fungus interactions. These fungi are predominantly Ascomycota, spanning a range of saprotrophic, endophytic and potentially pathogenic lifestyles, and include representatives of orders such as Helotiales, Hypocreales, Xylariales and Chaetothyriales, among others (Li et al., 2021b; Ma et al., 2015). The ecological roles of these fungi are likely more diverse than traditionally assumed, as many fungal taxa are now known to occupy dual ecological niches, functioning simultaneously as saprotrophs, endophytes or potential mycorrhizal partners depending on host and environmental context (Selosse et al., 2018). Some fungal groups commonly detected as root endophytes, including saprotrophic *Mycena* and members of Sebacinales, have been shown to also form mycorrhizal associations in orchids, suggesting that root endophytism may represent an evolutionary precursor to the orchid mycorrhizal habit (Selosse et al., 2010; Weiß et al., 2016; Selosse et al., 2018). There is growing evidence that ONF may facilitate germination and contribute to the growth and survival of mature plants, potentially interacting with OMF in ways that influence orchid fitness and population dynamics (Ma et al., 2015; Watkinson and Winkel, 2024). Understanding the full breadth of root-associated fungal diversity, including both mycorrhizal and non-mycorrhizal components, is therefore increasingly recognized as essential for a comprehensive view of orchid–fungus interactions and their conservation implications.

In Chile, there are 57 species of terrestrial orchids, grouped into eight genera (Rodríguez et al., 2018). They are distributed from the Tarapacá Region to the Magallanes Region (Rodríguez et al., 2018). Most species are endemic to the country, with *Chloraea* and *Gavilea* being the most diverse genus (Novoa et al., 2015; Rodríguez et al., 2018). In addition, hybrid formation has been reported in several native orchid species (Novoa et al., 2015; Verdugo Ramírez et al., 2015). In central Chile, anthropogenic threats such as land-use change, livestock grazing and forest fires affect the survival of several populations (Novillo and Ojeda, 2008; Atala et al., 2017). Furthermore, limited taxonomic and biological knowledge of many species hampers accurate assessments of their conservation status, which therefore remains unknown for many taxa (Correa, 1969; Novoa et al., 2015). Currently, 15 Chilean orchid species are classified under conservation threat categories (MMA, 2025), however, field observations indicate declines in many orchid populations, which could lead to serious conservation problems (Atala et al., 2017; Herrera et al., 2019).

*Chloraea* Lindl. has its center of diversity in southern South America (Arroyo et al., 1995), comprising approximately 27 species in Chile, distributed from the Coquimbo Region southwards (Rodríguez et al., 2018). Its species occupy a wide range of environments, from native forest understories to highly disturbed open sites (Correa, 1969; Novoa et al., 2015). Despite this ecological breadth, most species have not been adequately assessed in terms of conservation status (Novoa et al., 2015). Recent evaluations indicate that at least eight *Chloraea* species fall under some threat category (MMA, 2025), reinforcing the need for studies that support effective conservation strategies for Chilean orchids (Novoa et al., 2015). Many chilean orchid species depend on specific OMF for germination (Pereira et al., 2015, 2017, 2018, 2021; Mujica et al., 2021; Castillo-Novales et al., 2022; Herrera et al., 2019; Jeldes-Cajas et al., 2024). Nevertheless, knowledge of orchid–fungus interactions in *Chloraea* remains relatively scarce and is largely based on culture-dependent studies (Pereira et al., 2014, 2021; Atala et al., 2015; Herrera et al., 2019; Mujica et al., 2021), with the associated limitations stated above. In this context, the objective of this study is to evaluate the diversity and composition of root-associated fungal communities across species of the genus *Chloraea* along the central coast of Chile, and to explore whether fungal diversity and mycorrhizal specificity are associated with differences in geographic distributional range among species. Analysis of associated fungal communities will improve our understanding of their influence on key processes in the life cycle of orchids and provide relevant information for the conservation, especially *in situ* conservation, of this genus.

## Materials and methods

### Study sites

The study area encompasses the central coast of Chile, characterized by a coastal Mediterranean climate with moderate temperatures due to oceanic influence and an extended dry season during summer (Di Castri and Hajek, 1976). Mean annual precipitation ranges from 300 to 500 mm, concentrated mainly in winter. Soils across the study zone are predominantly sandy clay loam to loamy in texture, with a gradual transition to finer-textured soils southwards along the coastal range, consistent with the increasing precipitation gradient across the study zone (Dinamarca et al., 2023). A total of 21 sampling sites were surveyed along the central coast, spanning from the Valparaíso Region to the Biobío Region, from the shoreline to the coastal mountain range. All sampling sites correspond to coastal matorral, characterized by open shrublands and fragmented sclerophyllous forests dominated by *Baccharis* L., *Cryptocarya alba* (Molina) Looser, *Lithraea caustica* (Molina) Hook. and Arn., *Peumus boldus* Molina, *Puya* Molina, *Quillaja saponaria* Molina and *Schinus* L (Luebert and Pliscoff, 2017). No sampling site was located inside a native forest; however, *Pinus radiata* D.Don was present in the surrounding landscape of some sites, either adjacent to or within the sampling area, reflecting the widespread establishment of pine plantations along the central Chilean coast. Populations of 11 terrestrial orchid species of the genus *Chloraea* were sampled, representing 11 of the 16 species (close to 70%) described for central coastal Chile (Rodríguez et al., 2018): *Chloraea bletioides* Lindl., *Chloraea chrysantha* Poepp., *Chloraea chrysochlora* Phil., *Chloraea cristata* Lindl., *Chloraea disoides* Lindl., *Chloraea galeata* Lindl., *Chloraea gavilu* Lindl., *Chloraea heteroglossa* Rchb.f., *Chloraea lamellata* Lindl., *Chloraea multiflora* Lindl. and *Chloraea philippii* Rchb.f. Sampling was conducted during spring–summer, coinciding with the flowering period of each species. Individuals were identified in the field based on their distinctive floral morphology, following Novoa et al. (2015).

### Geographic range estimation

Occurrence records for all *Chloraea* species were compiled from ten Chilean herbaria (AGUCH, CONC, EIF, VALD, JBN, PUCV, SGO, SQF, ULS, VALPL) and supplemented by field records from the AEF laboratory. All available records for the genus were included regardless of the sampling range to obtain national-scale distributions and data formats were standardised across databases prior to analysis. Two complementary range metrics were calculated from these records. The extent of occurrence (EOO) was estimated using Minimum Convex Polygons (MCP), computed with the sp package in R (R Core Team, 2024) and recorded in km². The area of occupancy (AOO) was estimated by overlaying a 10 × 10 km grid onto occurrence records projected to UTM coordinates and counting only unique occupied cells. Latitudinal range was calculated as the absolute difference in decimal degrees between the northernmost and southernmost occurrence records per species. For *C. disoides*, northern (Valparaíso Region, ca. 33° S) and southern (Araucanía Region, ca. 38° S) populations were treated as separate entities to avoid overestimating the total range across the intervening unoccupied area.

### Root sample collection

Root segments were collected from individuals of the *Chloraea* species present at each site. One root per individual was collected from each *Chloraea* plant sampled at each site. *Chloraea* species are perennial geophytes whose root system persists across seasons (Steinfort et al., 2012); roots were therefore sampled during the active growing season (spring–summer), coinciding with the flowering period. The entire root was excised from base to tip. Only one root was excised from each plant to preserve the integrity of sampled individuals. All samples were kept on ice during transport and stored at −20 °C until DNA extraction.

### DNA extraction, PCR amplification and sequencing

Prior to DNA extraction, roots were surface-decontaminated by rinsing with distilled water, followed by repeated spraying with 96% ethanol until no soil was visibly present on the root surface, and a final rinse with distilled water. Every root, base to tip, was then homogenized in full prior to DNA extraction, without positional standardization along the root axis. Total DNA was extracted from 200 mg of root tissue per sample using the Quick-DNA™ Fecal/Soil Microbe Miniprep Kit (Zymo Research, Orange, CA, USA) following the manufacturer’s instructions. DNA concentration was verified with a TAKE3 Epoch micro-volume spectrophotometer (BioTek, San Francisco, CA, USA). DNA samples were sent to Novogene Co. Ltd. (Sacramento, CA, USA) for fungal ITS2 amplification, library preparation, and paired-end Illumina sequencing. Amplification was performed using primers ITS2-3F (5′-GCATCGATGAAGAACGCAGC-3′) and ITS2-4R (5′-TCCCTCCGCTTATTGATATGC-3′) (Op De Beeck et al., 2014). The ITS2-3F/ITS2-4R primer set was selected for two reasons: first, it targets the ITS2 subregion, which has been established as the universal fungal barcode and provides broad taxonomic coverage across the fungal kingdom, particularly Ascomycota and Basidiomycota (Op De Beeck et al., 2014; Nilsson et al., 2019), making it suitable for untargeted characterization of root-associated fungal communities; and second, its use (recommended by the Society for the Protection of Underground Networks, SPUN), ensures methodological consistency and comparability of fungal community data across a global sampling framework.

### Sequence processing and taxonomic assignment

Raw sequences were processed in Galaxy (v. 1.30.0) (Galaxy Community, 2024). Quality profiles of forward and reverse reads were inspected visually and quality filtering was carried out with DADA2 (Callahan et al., 2016), which removed primers, merged paired-end reads, corrected sequencing errors and eliminated chimeric and residual PhiX reads. Reads were dereplicated and amplicon sequence variants (ASVs) were inferred. ASVs, which Kauserud (2023) defines as conceptually equivalent to OTUs inferred through strict sequence agglomeration based on error models, were preferred over traditional OTU-based clustering approaches, as DADA2 has been shown to more accurately recover fungal community richness and composition compared to USEARCH- and VSEARCH-based pipelines in evaluations using synthetic communities (Pauvert et al., 2019), and has been adopted in recent metabarcoding studies of orchid root-associated fungal communities (Herrera et al., 2022; Weerasuriya et al., 2022). Furthermore, ASV labels are consistent and reproducible across studies, enabling future cross-study comparisons (Callahan et al., 2017). Taxonomic assignment was performed using a naïve Bayesian classifier (Wang et al., 2007) against the UNITE fungal ITS database (v. 8.0; Nilsson et al., 2019) with a confidence threshold of 97%. ASVs with relative abundance below 0.01% across all samples were removed (Beermann et al., 2018; Steinke et al., 2021). To address potential host plant DNA contamination, ASVs lacking complete taxonomic assignment were subjected to manual verification via BLAST against the NCBI nucleotide database (Camacho et al., 2009). ASVs matching orchid sequences with >95% identity and >90% coverage were classified as host plant contamination and removed from the dataset prior to all downstream analyses. Orchid mycorrhizal fungi (OMF) were identified among all assigned ASVs by filtering for taxa belonging to Ceratobasidiaceae, Sebacinaceae, Serendipitaceae and Tulasnellaceae (Dearnaley et al., 2012).

### Statistical analyses

Alpha diversity of root-associated fungal communities was characterised per orchid species using the Shannon (H’), Pielou (J’), Menhinick, inverse Simpson and observed ASV richness indices (mean ± SD). Differences in alpha diversity among species were evaluated with the Kruskal–Wallis test, restricted to species represented by more than one root sample. Beta diversity was estimated using Bray–Curtis dissimilarity and visualised by hierarchical clustering with average linkage (UPGMA), both at the level of individual samples and as mean pairwise dissimilarities between species. Differences in community composition among orchid species were tested with PERMANOVA (999 permutations). Orchid mycorrhizal fungi (OMF) were identified by filtering ASVs assigned to Ceratobasidiaceae, Sebacinaceae, Serendipitaceae and Tulasnellaceae. For each orchid species, the number of OMF-positive samples and the mean relative abundance of OMF reads were calculated; abundance was computed exclusively from OMF-positive samples. Linear regression analyses were performed between six alpha diversity indices (Shannon H’, Pielou J’, observed ASV richness, Menhinick index, inverse Simpson index, and OMF ASV richness) and three geographic range metrics (EOO, AOO, and latitudinal range), using log_10_-transformed values for EOO and AOO. Analyses were conducted for all 11 species and for the subset of eight species with more than one root sample (*C. galeata*, *C. heteroglossa*, and *C. philippii* excluded). All analyses and visualisations were performed in Python using the libraries pandas, scipy, scikit-bi and matplotlib.

## Results

### Geographic range estimation

A total of 1,795 occurrence records were compiled for the family Orchidaceae, of which 1,099 corresponded to the genus *Chloraea*, with 840 records assigned to identified species. 465 records belonged to the 11 study species (**Table 1**). Latitudinal range varied considerably among species, from 0.11° in *C. heteroglossa* to 11.17° in *C. chrysochlora*. Accordingly, *C. heteroglossa* also presented the smallest EOO and AOO (3.93 km² and 200 km², respectively), while *Chloraea galeata* exhibited the largest (106660 km² and 5000 km², respectively). For *C. disoides*, the northern population (n = 23) accounted for the majority of the total EOO, AOO, and latitudinal range (16995 km², 1400 km² and 2.30° respectively), while the southern population (n = 5) presented a markedly reduced values across all three metrics (192 km², 300 km² and 0.24°, respectively).

**Table 1:** Geographic range estimates by species.

Species	No. of records	EOO (km²)	AOO (km²)	Lat. range (°)
*C. bletioides*	70	44726	3700	4.54
*C. chrysantha*	66	77120	3900	6.02
*C. chrysochlora*	32	104094	2200	11.17
*C. cristata*	17	3059	900	0.91
*C. disoides*	28	17187	1700	2.54
*C. disoides* (North)	23	16994	1400	2.30
*C. disoides* (South)	5	192	300	0.24
*C. galeata*	83	106660	5000	7.38
*C. gavilu*	55	101804	3100	6.84
*C. heteroglossa*	5	3.93	200	0.11
*C. lamellata*	19	21830	1500	2.57
*C. multiflora*	37	51955	2200	5.75
*C. philippii*	10	21863	600	10.47

Number of occurrence records per species, with the extent of occurrence (EOO) estimated using Minimum Convex Polygons, the area of occupancy (AOO) calculated using a 10 × 10 km grid (both in km²), and the latitudinal range calculated as the difference between the northernmost and southernmost occurrence records (in decimal degrees). For *C. disoides*, the total EOO, AOO, and latitudinal range correspond to the sum of the northern and southern populations, which were treated as separate entities to avoid overestimating the range across the intervening unoccupied area.

### Sequence analysis

Prior to taxonomic filtering, the dataset comprised 10,197,132 reads across 11,385 ASVs. Of these, 6,334,385 reads (62.1%) and 6,185 ASVs (54.3%) were assigned to the host orchid and excluded from downstream analyses, yielding 3,862,747 fungal reads across 5,200 ASVs. The high proportion of host-assigned reads is consistent with the amplification of plant ITS2 sequences by general fungal primers when applied to root tissue.

**Figure 1:**
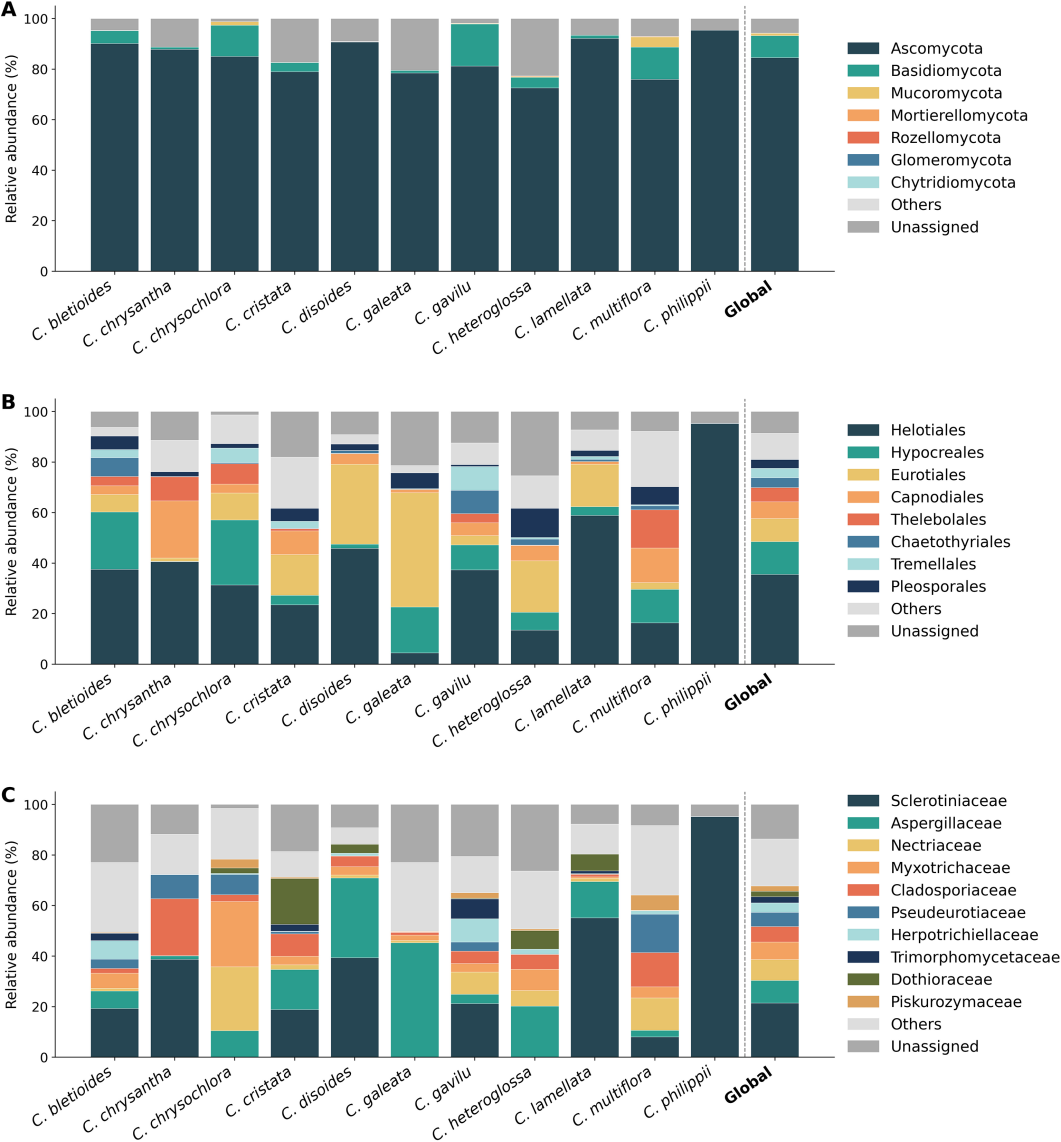
Taxonomic composition of root-associated fungal communities across *Chloraea* species at phylum **(A)**, order **(B)** and family **(C)** levels. Only the most abundant taxa are shown individually; remaining taxa are grouped as “Others”. Grey segments indicate unassigned reads.

Across all *Chloraea* root samples, Ascomycota was the dominant phylum, comprising 84.5% of total reads, followed by Basidiomycota (8.7%), unassigned sequences (5.8%) and Mucoromycota (<1%) (**Figure 1A**). Minor phyla were also detected at trace abundances: Mortierellomycota (<0.3%) was present in several species but absent from *C. chrysantha*, *C. galeata* and *C. philippii*; Glomeromycota (<0.1%) was detected in *C. bletioides*, *C. chrysochlora*, *C. cristata* and *C. galeata*; Chytridiomycota was restricted to *C. lamellata* and *C. multiflora*; Rozellomycota was detected only in *C. bletioides* and *C. multiflora*; Olpidiomycota only in *C. lamellata*; and Zoopagomycota only in *C. multiflora*. At the class level, Leotiomycetes was the most abundant class overall (41.2%), followed by Sordariomycetes (14.3%), Eurotiomycetes (13.1%), Dothideomycetes (12.8%) and Tremellomycetes (7.1%), the latter representing the most abundant Basidiomycota class (**Figure 1B**). At the family level, Sclerotiniaceae represented the most abundant family across all species (21.4%), followed by Aspergillaceae (9.0%), Nectriaceae (8.2%), Myxotrichaceae (7.0%) and Cladosporiaceae (6.1%). Within Basidiomycota, Trimorphomycetaceae (2.4%) and Piskurozymaceae (2.0%) were the most abundant families (**Figure 1C**).

Despite the shared dominance of Ascomycota, the relative contribution of major taxa varied considerably among *Chloraea* species. The proportion of Basidiomycota ranged from absent in *C. philippii* to 16.7% in *C. gavilu*, 12.8% in *C. multiflora* and *C. chrysochlora* (12.3%), while most remaining species harbored less than 5% Basidiomycota. At the class level, Leotiomycetes dominated in most species, reaching its highest relative abundance in *C. philippii* (95.3%) and *C. lamellata* (58.9%), whereas *C. cristata* and *C. heteroglossa* were instead dominated by Dothideomycetes (33.1% and 25.4%, respectively) and *C. galeata* by Eurotiomycetes (45.7%). At the family level, Sclerotiniaceae was consistently among the top families in most species, reaching 95.2% in *C. philippii* and 55.1% in *C. lamellata*, while *C. chrysochlora* was dominated by Myxotrichaceae (25.8%) and Nectriaceae (25.3%) and *C. multiflora* by Pseudeurotiaceae (15.1%) and Cladosporiaceae (13.6%). The proportion of unassigned reads was notably higher in *C. heteroglossa* (22.7%), *C. galeata* (20.5%) and *C. cristata* (17.3%) compared to the global mean (5.8%).

Alpha diversity of root-associated fungal communities varied considerably across *Chloraea* species (**Table 2**). Shannon index ranged from 0.45 (*C. philippii*) to 3.99 (*C. heteroglossa*), while Menhinick index ranged from 0.25 (*C. philippii*) to 1.40 (*C. heteroglossa*), and inverse Simpson from 1.14 (*C. philippii*) to 20.81 (*C. heteroglossa*). Among species with more than one sample, *C. cristata* showed the highest mean Shannon diversity (H’ = 3.12 ± 0.56) and Menhinick index (0.96 ± 0.21), followed by *C. multiflora* (H’ = 3.03 ± 1.02) and *C. gavilu* (H’ = 2.95 ± 0.66, Menhinick = 0.98 ± 0.36), while *C. chrysantha* presented the lowest values (H’ = 1.96 ± 0.54) and *C. chrysochlora* the lowest Menhinick (0.61 ± 0.35). For inverse Simpson, *C. lamellata* showed the highest mean value among species with more than one sample (12.81 ± 8.43), followed by *C. multiflora* (11.88 ± 6.85) and *C. gavilu* (9.49 ± 6.73), while *C. chrysantha* presented the lowest (2.76 ± 0.94). Despite this variation, no statistically significant differences in alpha diversity were detected among species (Kruskal-Wallis, H = 6.07, p = 0.53).

**Table 2:** Alpha diversity indices of root-associated fungal communities across *Chloraea* species.

Species	n	Observed ASVs	Shannon (H’)	Pielou (J’)	Menhinick	Inv. Simpson
*C. bletioides*	6	171.8 ± 76.8	2.65 ± 0.99	0.50 ± 0.16	0.96 ± 0.44	7.57 ± 6.54
*C. chrysantha*	3	102.9 ± 14.4	1.96 ± 0.54	0.42 ± 0.11	0.65 ± 0.22	2.76 ± 0.94
*C. chrysochlora*	3	135.1 ± 66.6	2.39 ± 1.03	0.46 ± 0.18	0.61 ± 0.35	6.83 ± 5.84
*C. cristata*	4	157.0 ± 12.1	3.12 ± 0.56	0.61 ± 0.11	1.09 ± 0.21	8.63 ± 3.78
*C. disoides*	5	115.3 ± 26.3	2.35 ± 0.84	0.49 ± 0.17	0.85 ± 0.42	5.47 ± 4.79
*C. galeata*	1	98.0	3.04	0.66	0.99	9.91
*C. gavilu*	5	183.5 ± 40.6	2.95 ± 0.66	0.54 ± 0.12	0.98 ± 0.36	9.49 ± 6.73
*C. heteroglossa*	1	210.9	3.99	0.74	1.40	20.81
*C. lamellate*	4	130.3 ± 51.5	2.87 ± 1.54	0.57 ± 0.29	1.07 ± 0.57	12.81 ± 8.43
*C. multiflora*	5	162.1 ± 62.1	3.03 ± 1.02	0.58 ± 0.16	0.89 ± 0.40	11.88 ± 6.85
*C. philippii*	1	50.4	0.45	0.11	0.25	1.14

Values represent the mean ± standard deviation across samples per species. Species with n = 1 are reported without standard deviation. No significant differences in alpha diversity were detected among species.

**Figure 2:**
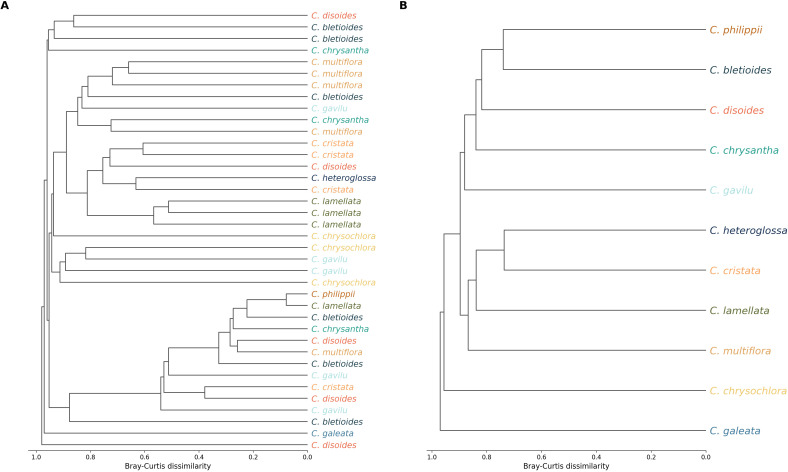
Hierarchical clustering of root-associated fungal communities of *Chloraea* species based on Bray-Curtis dissimilarity. **(A)** Clustering of all 38 individual samples. **(B)** Clustering based on mean pairwise Bray-Curtis dissimilarity between species.

On the other hand, beta diversity of root-associated fungal communities differed significantly among *Chloraea* species (PERMANOVA: pseudo-F = 1.37, p = 0.006). Overall mean Bray-Curtis dissimilarity across all 38 samples was 0.895 ± 0.157, indicating that fungal communities were largely distinct between individuals. Within-species dissimilarity was also consistently high across all species with more than one sample, ranging from 0.726 ± 0.063 in *C. cristata* to 0.950 ± 0.028 in *C. chrysantha* (**Figure 2A**).

At the species level, mean pairwise Bray-Curtis dissimilarity ranged from 0.736 to 1.000 across all species pairs (**Figure 2B**). The most similar pair was *C. cristata* and *C. heteroglossa* (BC = 0.736), followed by *C. cristata* and *C. lamellata* (BC = 0.813) and *C. heteroglossa* and *C. multiflora* (BC = 0.830). In contrast, *C. philippii* exhibited the highest dissimilarity values relative to most other species, reaching BC = 0.999 against *C. chrysochlora* and BC = 1.000 against *C. galeata*. *C. galeata* also showed consistently high dissimilarity values against the remaining species, with a mean inter-specific BC of 0.966 ± 0.016 across all pairs involving this species. *C. chrysochlora* presented similarly elevated dissimilarity values against all other species (mean = 0.960 ± 0.025), with the exception of its comparisons within the most dissimilar pairs.

A total of 21 OMF ASVs were identified out of 5,200 total ASVs, representing 0.40% of all ASVs and 0.32% of total reads. Of the four OMF families included in the analysis, Tulasnellaceae was not detected in any sample. Ceratobasidiaceae was the most represented family, comprising 17 ASVs and 5,883 reads, followed by Serendipitaceae (3 ASVs) and Sebacinaceae (2 ASVs). OMF were detected in 22 of 38 samples (57.9%), distributed across 8 of the 11 *Chloraea* species studied (**Table 3**).

**Table 3:** Orchid mycorrhizal fungi (OMF) detected in root samples of *Chloraea* species.

Species	n	Samples with OMF	OMF rel. abund. (%)	Ceratobasidiaceae	Sebacinaceae	Serendipitaceae
*C. bletioides*	6	4	1.36 ± 2.58	4	1	1
*C. chrysantha*	3	1	0.28	1	0	0
*C. chrysochlora*	3	0	–	0	0	0
*C. cristata*	4	0	–	0	0	0
*C. disoides*	5	5	0.08 ± 0.08	4	0	1
*C. galeata*	1	1	0.14	1	0	0
*C. gavilu*	5	4	0.07 ± 0.10	3	1	0
*C. heteroglossa*	1	1	2.45	1	0	0
*C. lamellate*	4	0	–	0	0	0
*C. multiflora*	5	5	2.24 ± 2.46	5	0	2
*C. philippii*	1	1	0.02	1	0	0
*Global*	38	22	0.92 ± 1.75	20	2	4

Columns indicate the number of samples in which each OMF family was detected. OMF relative abundance (%) represents the mean ± standard deviation calculated from OMF-positive samples only; (-) indicates no OMF were detected. Tulasnellaceae was included in the analysis but was not detected in any sample.

*C. bletioides* harbored 4 OMF ASVs: two belonging to Ceratobasidiaceae, one to Serendipitaceae (*Serendipita vermifera*) and one to Sebacinaceae (*Sebacina* sp.). *C. disoides* harbored 3 OMF ASVs, two Ceratobasidiaceae and one Serendipitaceae (*Serendipita indica*). *C. gavilu* harbored 5 OMF ASVs, all belonging to Ceratobasidiaceae except one Sebacinaceae (*Chaetospermum* sp.). *C. multiflora* harbored 4 OMF ASVs: three Ceratobasidiaceae (one of them *Thanatephorus cucumeris*) and one Serendipitaceae (*Serendipita vermifera*). *C. chrysantha, C. galeata, C. heteroglossa* and *C. philippii* each presented a single ASV, corresponding to Ceratobasidiaceae. No OMF ASVs were detected in *C. chrysochlora*, *C. cristata*, or *C. lamellata*. Notably, ASV_00078 (Ceratobasidiaceae) was the most abundant OMF ASV overall and was shared across six orchid species (*C. bletioides*, *C. disoides*, *C. galeata*, *C. gavilu*, *C. heteroglossa* and *C. multiflora*.).

**Figure 3:**
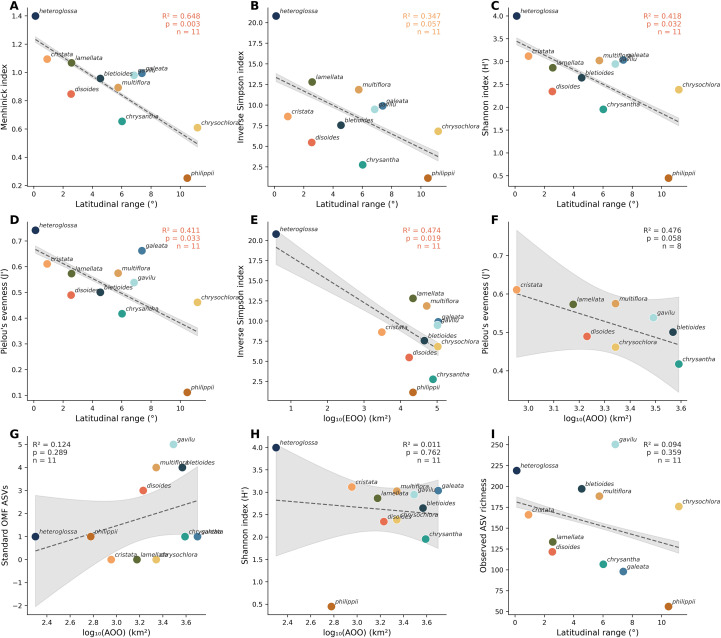
Relationships between fungal alpha diversity indices and geographic range metrics across *Chloraea* species. Panels **(A–D)** show Menhinick index **(A)**, inverse Simpson index **(B)**, Shannon index H’ **(C)**, and Pielou’s evenness J’ **(D)** plotted against latitudinal range (°). Panel **(E)** shows inverse Simpson index against log_10_-transformed EOO (km²). Panels **(F–I)** show Pielou’s evenness J’ **(F)**, standard OMF ASV richness **(G)**, Shannon index H’ **(H)**, and observed ASV richness **(I)** plotted against log_10_-transformed AOO (km²). Panel **(F)** excludes species represented by a single root sample (*C. galeata*, *C. heteroglossa*, and *C. philippii*; n = 8); all other panels include all 11 species. Dashed lines represent linear regression fits with 95% confidence intervals (shaded areas). Significant relationships (p < 0.05) are indicated in red.

No significant relationships were detected between any diversity index and AOO under either dataset, with the exception of a marginally non-significant association between Pielou and AOO when single-sample species were excluded (R² = 0.476, p = 0.058). For EOO, a significant negative relationship was detected with inverse Simpson when all 11 species were included (R² = 0.474, p = 0.019); Menhinick also approached significance under the same conditions (R² = 0.341, p = 0.059). All other EOO combinations were non-significant (R² < 0.25, p > 0.10). Latitudinal range showed significant negative relationships with Menhinick (R² = 0.594, p = 0.006), inverse Simpson (R² = 0.549, p = 0.009), Shannon (R² = 0.406, p = 0.035), and Pielou (R² = 0.377, p = 0.044) when all 11 species were included. These relationships were not significant when single-sample species were excluded (all p > 0.17). Notably, *C. heteroglossa* and *C. philippii* represent the minimum and maximum values of latitudinal range and Shannon index across the dataset, respectively (**Figure 3**).

## Discussion

In this study, we characterized the root-associated fungal diversity of eleven terrestrial *Chloraea* species across the central Chilean coast using an untargeted metabarcoding approach applied directly to field-collected root samples, without prior *in vitro* cultivation or OMF-specific primers. Our results revealed a compositionally diverse and highly variable fungal consortium dominated by Ascomycota, with OMF representing only a minor fraction of total root-associated diversity and a consistent negative relationship between fungal alpha diversity and geographic range. To our knowledge, this is the first study to apply such an approach to terrestrial orchids in Chile, providing a broader view of root-associated fungal assemblages than culture-dependent or targeted methods, which inherently capture only a subset of the fungi present (Herrera et al., 2019; Li et al., 2021b).

The dominance of Ascomycota in root-associated communities of *Chloraea* is consistent with patterns reported previously, orchids, like many other plants, also interact with fungi of this phylum with much greater diversity and frequency than those of OMF (Sudheep and Sridhar, 2012; Ma et al., 2015). The main orders are typically the Chaetothyriales, Capnodiales, Hypocreales, Helotiales and Xylariales, which have been reported in association with tropical and temperate orchids (Bayman et al., 1997; Dearnaley et al., 2012; Oliveira et al., 2014; Govinda Rajulu et al., 2016; Jacquemyn et al., 2016; Beltrán-Nambo et al., 2018; Novotná et al., 2018). Consistent with this, Helotiales, Chaetothyriales, Hypocreales and Capnodiales were detected in virtually all root samples (**Figure 1**) and Xylariales was present in only a minority of samples. Genera such as *Fusarium* and *Trichoderma* (order Hypocreales) were among the most widely distributed non-OMF fungi across *Chloraea* root samples (**Figure 1**), consistent with their frequent detection as root endophytes in both tropical and temperate orchids (Martos et al., 2012; Tao et al., 2013; Sufaati et al., 2016; Salazar-Cerezo et al., 2018; Sisti et al., 2019; Sarsaiya et al., 2020), where they act not as pathogens but as endophytes with potential growth-promoting effects on their hosts (Vujanovic et al., 2000; Bayman and Otero, 2006; Rodriguez et al., 2009; Selosse et al., 2018; Li et al., 2021b). Within Helotiales, Hyaloscyphaceae was detected in six of the eleven sampled *Chloraea* species (*C. gavilu*, *C. bletioides*, *C. lamellata*, *C. disoides*, *C. cristata* and *C. multiflora*). Members of this fungi family are known to adopt multiple symbiotic lifestyles, forming ericoid mycorrhizae, ectomycorrhizae, and endophytic associations, including with orchid roots (Vohník et al., 2022). The dominant genus within this family was *Gyoerffyella*, primarily described from freshwater aquatic habitats (Chauvet et al., 2016), though strains have also been isolated from ectomycorrhizal roots of *Picea abies* (Vohník et al., 2013). Its consistent detection across multiple *Chloraea* species may reflect the seasonal humidity of Mediterranean coastal soils during the sampling period, though its functional role in orchid roots remains unknown. The ecological and physiological roles of non-OMF fungi in orchid roots remain poorly understood, yet their frequent occurrence across species and sites suggests they may contribute to host fitness beyond the strictly mycorrhizal interaction, for instance by enhancing abiotic stress tolerance or nutrient acquisition (Ma et al., 2015).

Beyond endophytic Ascomycota, several ectomycorrhizal (ECM) families were detected at low but consistent abundances in *Chloraea* root samples, including Atheliaceae, Rhizopogonaceae, Thelephoraceae and Russulaceae. These families are not canonical orchid mycorrhizal partners, yet their detection in orchid root tissue is ecologically plausible in the context of this study, given the presence of *Pinus radiata* and native sclerophyllous trees at several sampling sites. ECM fungi typically form extensive mycelial networks that extend well beyond the root systems of their primary hosts, and it has been proposed that terrestrial orchids may opportunistically colonise or passively intercept these networks (Selosse et al., 2002; Bidartondo et al., 2004). Whether these interactions are ecologically functional or represent incidental contact warrants further investigation.

Basidiomycetous yeasts were also detected across nearly all sampled *Chloraea* species, representing one of the most broadly distributed fungal groups in the dataset. Three yeast families were consistently present: Piskurozymaceae (*Solicoccozyma* and *Piskurozyma*; detected in 10 of 11 species), Trimorphomycetaceae (*Saitozyma*; 8 of 11 species) and Bulleribasidiaceae (*Vishniacozyma*; 8 of 11 species). These genera are characteristic components of temperate forest soil and litter communities (Mašínová et al., 2017), yet their occurrence in orchid roots has received little attention and their functional role in this context remains completely unknown.

Up to one fifth of ASVs could not be assigned taxonomically against the UNITE database (**Figure 1**), a pattern that likely reflects the underrepresentation of local fungal taxa from biodiversity-rich and understudied regions such as central Chile in global reference databases (Marín et al., 2021; Quinteros-Urquieta et al., 2024). This geographic bias, skewed towards Northern Hemisphere ecosystems, has been previously recognized as a major limitation in fungal metabarcoding studies (Nilsson et al., 2019). In the context of terrestrial orchids, the unassigned fraction is particularly concerning because OMF lineages such as Tulasnellaceae and Ceratobasidiaceae tend to establish highly specific associations with their hosts and these lineages may be geographically unique, with local variants that lack representation in global databases (Dearnaley et al., 2012). This specificity reflects a coevolutionary history adapted to local ecological conditions, which can vary significantly even among neighboring populations of the same orchid species (Jacquemyn et al., 2017). Consequently, the true diversity of OMF associated with Chilean terrestrial orchids is likely underestimated. Beyond OMF, root-associated fungal communities of terrestrial orchids encompass a broad functional spectrum, saprotrophs, endophytes and potential pathogens, all of which are shaped by local environmental conditions including soil composition, biotic interactions and climate (Brundrett, 2017). Many of these taxa remain poorly described or unknown to science and the dual role of endophytic fungi as mutualists or opportunists, depending on host and environmental context, adds further complexity to their taxonomic and ecological characterization (Tedersoo et al., 2014).

The low relative abundance of OMF detected across *Chloraea* root samples (**Table 3**) and the complete absence of Tulasnellaceae, one of the most commonly reported OMF families in terrestrial orchids globally, are likely attributable, at least in part, to primer bias. The ITS2-3F/ITS2-4R primers used in this study target the ITS2 subregion, which, while preferred for its higher phylogenetic resolution and ASV yield compared to ITS1 (Mello et al., 2011; Tedersoo and Lindahl, 2016; Nilsson et al., 2019), is known to underperform in amplifying the most common OMF taxa due to accelerated rDNA evolution in these lineages (Taylor et al., 2002; Binder et al., 2005). OMF-specific primers such as ITS1-OF/ITS4-OF (Taylor and McCormick, 2008) or ITS86F/ITS4 (Waud et al., 2014) have been developed precisely to address this limitation, though the latter do not always perform well on soil samples (Liu et al., 2021). Future studies targeting OMF diversity in Chilean terrestrial orchids should carefully evaluate primer choice and its potential biases. Beyond primer limitations, the absence of OMF in some species and samples may also reflect genuine biological variation, as fungal associations in orchids are known to shift with ontogeny and phenological stage (McCormick et al., 2006; Rasmussen and Rasmussen, 2009). For instance, *Anacamptis morio* subsp. *champagneuxii* shows peak fungal diversity during flowering (Herrera-Rus et al., 2020) and unpublished data from our group suggest that *C. lamellata* and *C. chrysochlora* modify their mycobiont assemblages across ontogenetic and phenological stages, with younger root segments lacking OMF compared to older ones (Pereira et al., 2023). It should also be noted that, while DADA2-inferred ASVs have been widely adopted in fungal metabarcoding studies, their use for ITS2 data may capture intraspecific sequence variation, potentially bloating absolute ASV richness estimates for some fungal groups (Tedersoo et al., 2022; Kauserud, 2023). Absolute alpha diversity values reported here should therefore be interpreted with this caveat in mind. However, community composition and comparative richness patterns across species are considered robust to this methodological choice, as both ASV and OTU methods have been shown to recover consistent community structure patterns (Kyaschenko et al., 2026; Joos et al., 2020).

Among the *Chloraea* species studied, *C. cristata* stood out for harboring the most internally consistent root-associated fungal communities. Within-species Bray-Curtis dissimilarity for this species was significantly lower than that of the remaining species (p = 0.0016), indicating that individuals share a similar fungal assemblage regardless of sampling site. No OMF were detected in any of its root samples (**Table 3**), however, given both the primer limitations discussed above and the disproportionately high proportion of unassigned sequences in this species (**Figure 1**), the presence of a key OMF cannot be excluded. This is, to our knowledge, the first study to characterize the root-associated fungal community of this vulnerable species (DS 41/2011, Ministerio del Medio Ambiente) and confirming its apparent fungal specificity will require sampling additional populations and conducting symbiotic germination trials with its associated fungi.

In contrast, *C. disoides* exhibited high within-species variability in its overall root-associated fungal community (**Figure 2**), suggesting that individuals associate with broadly different fungal assemblages depending on site. Nevertheless, a consistent pattern emerged at the OMF level, Ceratobasidiaceae was the only OMF family detected across all populations, present in both Valparaíso localities (**Table 3**), despite the two Ceratobasidiaceae ASVs involved being clearly distinct sequences (88.98% pairwise identity), indicating association with different taxa within the same family rather than a shared strain. *Serendipita indica*, a root-colonizing endophyte, originally described from orchids in the Thar desert in India (Verma et al., 1998) and known to promote plant growth across a broad host range (Waller et al., 2005; Serfling et al., 2007), was detected exclusively in the Araucanía population. This geographically structured pattern of OMF association, with family-level fidelity to Ceratobasidiaceae across a disjunct distribution, suggests that *C. disoides* may exhibit specificity at the family rather than species level, a pattern that warrants further investigation through targeted culture-based approaches and symbiotic germination trials, and for which preliminary evidence from culture-based studies in disjunct populations provides initial support (Pereira et al., 2024). This species is currently classified as critically endangered (DS 41/2011, Ministerio del Medio Ambiente) and understanding the geographic structure of its mycorrhizal associations has direct implications for any future conservation or reintroduction programme.

The distribution of orchids is significantly influenced by the availability of compatible fungi, which are essential for seed germination and population establishment (McCormick et al., 2018; Li et al., 2021b). To evaluate whether fungal diversity in roots is associated with geographic range in *Chloraea*, we tested the relationship between multiple alpha diversity indices and geographic range metrics across species. Contrary to expectations, a consistent negative relationship was detected between fungal species richness and latitudinal range (**Figure 3**), indicating that species with broader distributions tend to associate with less diverse root-associated fungal communities. Beyond richness, evenness indices also showed a negative trend with latitudinal range (**Figure 3**), suggesting that widely distributed species not only associate with fewer fungal taxa but also harbor communities dominated by one or a few abundant taxa. Consistent with this, an unidentified Sclerotiniaceae was the single most abundant fungal taxon in the three most widely distributed species, *C. bletioides*, *C. gavilu* and *C. multiflora*, alongside *Ilyonectria* spp. (Nectriaceae), both belonging to groups that include widely distributed taxa commonly reported as root endophytes across temperate and Mediterranean ecosystems (**Figure 1**). This pattern, while requiring validation with broader sampling, is consistent with the hypothesis that widely distributed orchid species may achieve their broad ranges partly through association with a reduced set of ubiquitous and cosmopolitan fungal taxa, rather than through fungal generalism, a mechanism previously documented in the mycoheterotrophic orchid *Eulophia zollingeri*, which maintains wide geographic distribution through high specificity toward a single widespread fungal partner (Ogura-Tsujita and Yukawa, 2008). This result contrasts with findings for the related Chilean genus *Bipinnula*, where range-restricted species showed significantly lower mycorrhizal richness than widespread ones (Claro et al., 2020), and points to a more complex relationship between fungal diversity and distributional breadth in *Chloraea* that warrants further investigation combining fungal community data with information on the geographic distribution of the associated fungal taxa.

Understanding the degree of mycorrhizal specificity in *Chloraea* species has direct implications for their conservation and long-term management. Species with apparently narrow fungal associations, such as *C. cristata* and *C. heteroglossa*, may be particularly vulnerable to environmental changes that reduce the abundance or availability of their compatible mycobionts, compounding the threats already posed by their restricted distributions and small population sizes. For these species, *in situ* conservation strategies are preferable over *ex situ* approaches, as they preserve not only the orchid populations but also the soil fungal communities on which they depend (Batty et al., 2002; Swarts et al., 2010). Future studies should also integrate soil fungal community data from the same sampling sites, as understanding the relationship between soil fungal availability and root colonisation patterns is essential for a comprehensive interpretation of mycorrhizal specificity and its role in determining orchid distribution ranges. Indeed, the high mycorrhizal specificity observed in some Chilean *Chloraea* species suggests that reintroduction programmes that do not account for the presence of compatible OMF are unlikely to succeed in the long term (Ramsay and Dixon, 2003; Claro et al., 2020). More broadly, given that root-associated fungal communities extend well beyond OMF, rhizosphere microbiome management approaches may prove more effective than focusing on individual fungal taxa in isolation. This is especially urgent in central Chile, which harbours the greatest diversity of *Chloraea* yet has less than 1% of its territory included within the state protected area network (Cádiz-Véliz et al., 2023), leaving most populations and their associated fungal communities outside any formal conservation framework.

Together, our findings reveal that root-associated fungal communities of Chilean terrestrial orchids are far more diverse and compositionally variable than previously appreciated from culture-based studies, encompassing not only OMF but a broad spectrum of endophytic and saprotrophic taxa whose ecological roles remain largely unexplored. The contrasting patterns observed across *Chloraea* species, from the internally consistent assemblages of *C. cristata* to the geographically structured OMF fidelity of *C. disoides*, illustrate that mycorrhizal specificity varies markedly even within a single genus, with potential consequences for distributional breadth and vulnerability to habitat loss. Advancing our understanding of these interactions will require combining untargeted and OMF-specific sequencing approaches, expanding sampling across populations and phenological stages and investing in local fungal reference databases that better represent the Southern Hemisphere’s unique mycological diversity. Only by integrating this knowledge into conservation planning can we hope to develop effective strategies for the long-term persistence of Chile’s threatened terrestrial orchids.

## Data Availability

The sequence data generated in this study have been deposited at DDBJ/EMBL/GenBank as a Targeted Locus Study under accession number KJLX00000000 (version KJLX01000000), associated with BioProject PRJNA1474502.
